# Pulmonary Arterial Hypertension: From Molecular Basis to Therapeutic Approaches

**DOI:** 10.3390/biomedicines14030705

**Published:** 2026-03-18

**Authors:** Rui Adão

**Affiliations:** 1 Department of Pharmacology and Toxicology, School of Medicine, Universidad Complutense de Madrid, 28040 Madrid, Spain; rdacosta@ucm.es; 2 Centro de Investigación Biomédica en Red de Enfermedades Respiratorias (CIBERES), 28029 Madrid, Spain; 3 Instituto de Investigación Sanitaria Gregorio Marañón (IiSGM), 28007 Madrid, Spain

Pulmonary arterial hypertension (PAH) is a progressive disorder characterized by pathological remodeling of the pulmonary vasculature, ultimately leading to right heart failure and premature death [1]. It is a rare disease, with an estimated prevalence of approximately 1 in 67,000 individuals (ORPHA: 182090). Despite significant therapeutic advances over recent decades, the five-year survival remains limited at around 60% [2]. This underscores the urgent need to identify novel biomarkers, uncover innovative therapeutic targets, and develop more effective treatment strategies to improve patient outcomes. This Special Issue of *Biomedicines* brings together a diverse collection of original research and review articles that collectively advance our understanding of PAH from molecular mechanisms to potential therapeutic strategies. Collectively, these articles illustrate the diverse and multidisciplinary nature of PAH research, from novel computational diagnostics to metabolomic insights into systemic effects of disease and the clinical optimization of therapy.

In total, six papers were included in this Special Issue (four articles and two reviews) [3,4,5,6,7,8]. The contributions are listed below:

In contribution 1, Jara et al. [3] presents a novel and clinically relevant approach to the non-invasive estimation of pulmonary arterial pressure. By integrating clinical 4D Flow MRI data with a reduced-order Navier–Stokes model within a physics-informed neural network (PINN) framework, this study shows that essential hemodynamic variables (including pressure, velocity, and arterial area) can be accurately predicted without dependence on invasive catheterization [3]. Importantly, the model pressure estimates are consistent with the established physiological ranges [9], highlighting the potential of PINNs to complement traditional diagnostic techniques and support future validation in larger patient cohorts. However, in future, it would be essential to validate the model in larger populations and confirm pulmonary hypertension cases diagnosed through catheterization. This approach may have significant implications for PAH, offering a promising non-invasive tool for earlier detection, improved risk stratification, and longitudinal monitoring of disease progression.

In contribution 2, Shafeghat et al. [4] provides an updated and integrative overview of PAH, with particular emphasis on right ventricular (RV) adaptation and failure as the central determinant of prognosis. The authors detail the molecular and cellular mechanisms driving pulmonary vascular remodeling while underscoring the pivotal role of RV–pulmonary artery coupling in disease development. Importantly, the review highlights improvements in multimodality imaging for the assessment of RV structure and function, as well as existing and emerging therapeutic strategies aimed at preserving or restoring RV performance [4]. By placing the RV at the core of PAH pathobiology and management, this work reinforces the concept that targeting RV function is essential for improving clinical outcomes and guiding future translational research [10].

In contribution 3, Wawrzyniak et al. [5] explore sex-specific metabolic signatures in PAH using a comprehensive multiplatform plasma metabolomics methodology. By identifying distinct metabolic profiles related with either male or female PAH patients, the authors provide new insights into the biological underpinnings that may contribute to known sex differences in the disease progression and therapeutic response. Notably, this work underscores the potential of metabolomic biomarkers to enhance personalized risk stratification and to inform tailored therapy strategies [5]. These findings pave the way for more individualized PAH management, highlighting the need to consider gender-specific metabolic pathways in prognosis [11], therapeutic decision-making, and potential precision medicine programs.

In contribution 4, Mansoor and Ibrahim [6] provide an up-to-date and clinically centered analysis of the evolving understanding of RV dysfunction in PAH. The authors integrate mechanistic insights into RV maladaptation with current and emerging therapeutic strategies specifically aimed at preserving or restoring RV function. By studying new molecular targets, metabolic modulators, and interventions that directly affect RV performance and RV–pulmonary vascular coupling, this review highlights new directions that extend beyond conventional vasodilator therapy [6]. Clinically, the review emphasizes that improving RV function, rather than reducing pulmonary vascular resistance, is essential to enhance exercise capacity, reduce clinical worsening, and improve survival in PAH patients [12], underscoring the promise of next-generation therapy pathways.

In contribution 5, Assis et al. [7] investigate the systemic consequences of PAH beyond the cardiopulmonary axis by examining its impact on male reproductive organs in a rodent model. The study demonstrates that PAH induces significant alterations in testicular and epididymal structure and function; significantly, a regimen of combined physical training partially attenuates these alterations [7]. By highlighting the interplay between chronic cardiopulmonary stress and reproductive health, this work broadens our understanding of PAH multisystem effects. These findings suggest that structured physical exercise may offer protective benefits against PAH-related reproductive dysfunction, underscoring the potential value of incorporating tailored exercise programs into holistic management strategies for PAH patients [13].

In contribution 6, Kędzierski et al. [8] study the therapeutic implications of systematic dose escalation of treprostinil in PAH patients. By analyzing the hemodynamic responses, symptom routes, and safety effects across a range of dosing programs, the authors underscore that individualized and carefully titrated dose escalation can significantly enhance the clinical status and functional capacity. The study highlights the balance between optimizing vasodilatory and anti-remodeling effects while maintaining tolerability, reinforcing the need for tailored therapy strategies in complex PAH management [8]. At the clinical level, these findings support a proactive dose escalation method with treprostinil to enhance the hemodynamic benefit and improve patient-centered outcomes, highlighting the value of accuracy in dosing in current PAH therapy [14].

Collectively, the studies in this Special Issue highlight recent advances in PAH research, from innovative diagnostics (contribution 1) and multi-omics profiling (contribution 3) to RV assessment (contributions 2 and 4) and optimized therapeutic strategies (contributions 5 and 6). By bringing together multidisciplinary and translational perspectives, this Special Issue features the importance of integrating mechanistic insight with clinical innovation to progress the field of PAH.

## References

[1] Humbert M., Kovacs G., Hoeper M.M., Badagliacca R., Berger R.M.F., Brida M., Carlsen J., Coats A.J.S., Escribano-Subias P., Ferrari P. (2023). 2022 ESC/ERS Guidelines for the diagnosis and treatment of pulmonary hypertension. Eur. Heart J..

[2] Ruopp N.F., Cockrill B.A. (2022). Diagnosis and Treatment of Pulmonary Arterial Hypertension: A Review. JAMA.

[3] Jara S., Sotelo J., Ortiz-Puerta D., Estevez P.A., Uribe S., Chabert S., Salas R. (2025). Physics-Informed Neural Network for Modeling the Pulmonary Artery Blood Pressure from Magnetic Resonance Images: A Reduced-Order Navier-Stokes Model. Biomedicines.

[4] Shafeghat M., Raza Y., Catania R., Rahsepar A.A., Tilkens B., Cuttica M.J., Freed B.H., Dai J., Zhao Y.Y., Carr J.C. (2025). State of the Art in Pulmonary Arterial Hypertension: Molecular Basis, Imaging Modalities, and Right Heart Failure Treatment. Biomedicines.

[5] Wawrzyniak R., Gaillard T., Biesemans M., Zieba B., Lewicka E., Markuszewski M., Dabrowska-Kugacka A. (2025). Plasma Multiplatform Metabolomics Towards Evaluation of Gender Differences in Pulmonary Arterial Hypertension-A Pilot Study. Biomedicines.

[6] Mansoor M., Ibrahim A.F. (2025). Emerging Mechanistic Insights and Therapeutic Strategies for Pulmonary Arterial Hypertension: A Focus on Right Ventricular Dysfunction and Novel Treatment Pathways. Biomedicines.

[7] Assis M.Q., Leite L.B., Guimaraes-Ervilha L.O., Adao R., Reis E.C.C., Natali A.J., Machado-Neves M. (2025). Pulmonary Arterial Hypertension-Induced Reproductive Damage: Effects of Combined Physical Training on Testicular and Epididymal Parameters in Rats. Biomedicines.

[8] Kedzierski P., Banaszkiewicz M., Florczyk M., Pilka M., Manczak R., Wieteska-Milek M., Szwed P., Kasperowicz K., Wrona K., Darocha S. (2025). The Importance of Dose Escalation in the Treatment of Pulmonary Arterial Hypertension with Treprostinil. Biomedicines.

[9] Schafer M., Barker A.J., Kheyfets V., Stenmark K.R., Crapo J., Yeager M.E., Truong U., Buckner J.K., Fenster B.E., Hunter K.S. (2017). Helicity and Vorticity of Pulmonary Arterial Flow in Patients with Pulmonary Hypertension: Quantitative Analysis of Flow Formations. J. Am. Heart Assoc..

[10] Balsa A., Adao R., Bras-Silva C. (2023). Therapeutic Approaches in Pulmonary Arterial Hypertension with Beneficial Effects on Right Ventricular Function-Preclinical Studies. Int. J. Mol. Sci..

[11] Pagnesi M., Riccardi M., Savonitto G., Ameri P., Monti S., Driussi M., Gentile P., Specchia C., Oriecuia C., Adamo M. (2025). Sex differences in pulmonary arterial hypertension: Insights from the FOCUS-PAH registry. Int. J. Cardiol..

[12] Kiyota T., Mendes P., Pereira M.C. (2026). The role of the right ventricle in pulmonary arterial hypertension and imaging methods for non-invasive evaluation. Expert Rev. Respir. Med..

[13] Waller L., Kruger K., Conrad K., Weiss A., Alack K. (2020). Effects of Different Types of Exercise Training on Pulmonary Arterial Hypertension: A Systematic Review. J. Clin. Med..

[14] Kelly N.J., Chan S.Y. (2022). Pulmonary Arterial Hypertension: Emerging Principles of Precision Medicine across Basic Science to Clinical Practice. Rev. Cardiovasc. Med..

